# The effect of induced optimism on early pain processing: indication by contact heat evoked potentials (CHEPs) and the sympathetic skin response (SSR)

**DOI:** 10.1093/scan/nsad042

**Published:** 2023-09-01

**Authors:** Johanna Basten-Günther, Laura Jutz, Madelon L Peters, Janosch A Priebe, Stefan Lautenbacher

**Affiliations:** Department of Physiological Psychology, University of Bamberg, Bamberg 97047, Germany; Department of Physiological Psychology, University of Bamberg, Bamberg 97047, Germany; Department of Clinical Psychological Science, Maastricht University, Maastricht 6200 MD, The Netherlands; Department of Physiological Psychology, University of Bamberg, Bamberg 97047, Germany; Center of Interdisciplinary Pain Medicine, Department of Neurology, Klinikum Rechts der Isar, Technical University of Munich, Munich 81675, Germany; Department of Physiological Psychology, University of Bamberg, Bamberg 97047, Germany

**Keywords:** pain, pain-evoked potentials, EEG, optimism, resilience

## Abstract

Situationally induced optimism has been shown to influence several components of experimental pain. The aim of the present study was to enlarge these findings for the first time to the earliest components of the pain response by measuring contact heat evoked potentials (CHEPs) and the sympathetic skin response (SSR). Forty-seven healthy participants underwent two blocks of phasic thermal stimulation. CHEPs, the SSR and self-report pain ratings were recorded. Between the blocks of stimulation, the ‘Best Possible Self’ imagery and writing task was performed to induce situational optimism. The optimism manipulation was successful in increasing state optimism. It did, however, neither affect pain-evoked potentials nor the SSR nor self-report pain ratings. These results suggest that optimism does not alter early responses to pain. The higher-level cognitive processes involved in optimistic thinking might only act on later stages of pain processing. Therefore, more research is needed targeting different time frames of stimulus processing and response measures for early and late pain processing in parallel.

## Introduction

Optimism is commonly defined as generalized positive expectancies concerning the future (Scheier and Carver, 1985). Among other positive health-related outcomes, optimism is reportedly related to less experimental and less acute and chronic clinical pain (for overviews, see Garofalo, 2000; Goodin and Bulls, 2013; Basten-Guenther *et al.*, 2019). Thus, it may be a pain resilience factor, i.e. a pain-protecting personal characteristic.

To establish a causal link, an experimental manipulation of optimism is essential. The Best Possible Self (BPS) imagery and writing task (King, 2001) has repeatedly and successfully been used to induce state optimism (Peters *et al.*, 2010; Hanssen *et al.*, 2013; Boselie, *et al.*, 2014; Basten‐Günther *et al.*, 2021), leading to lower pain ratings (Hanssen *et al.*, 2013, but not in Basten‐Günther *et al.*, 2021; Boselie *et al.*, 2014), as well as to changes in facial expression of pain, attentional preference and executive functioning (Boselie *et al.*, 2014; Peters *et al.*, 2016; Basten‐Günther *et al.*, 2021).

According to the four-stage model of pain processing proposed by Price (1988) and Wade *et al.* (1992), the earliest stage in pain processing comprises the sensory-discriminative dimension. As a second stage, immediate unpleasantness is added. Suffering and first elements of pain behaviour are integrated into stages 3 and 4 (Wade *et al.*, 1996).

Based on this model, the present study aims at examining for the first time the effect of optimism on the earliest stages of pain processing. For this reason, nociceptive-evoked brain potentials and the sympathetic skin response (SSR) are recorded (Price and Bushnell, 2004).

To evoke nociceptive potentials, contact heat was applied. Phasic stimuli with a steep onset and of a short duration have been established as preferable for measuring early nociceptive potentials, whereas tonic stimuli might be more useful for tapping later stages of pain processing (Kobal *et al.*, 1994). The second negative (N2) and second positive (P2) peaks after stimulus onset were measured as contact heat evoked potentials (CHEPs). These, as well as their difference, the N2P2 complex, reflect early sensory processing which precedes more elaborate cognitive appraisal (Chen *et al.*, 2006; Granovsky *et al.*, 2008). CHEPs are a well-established psychophysiological pain measure providing nociceptive specificity and high diagnostic accuracy (Granovsky *et al.*, 2008; Di Stefano *et al.*, 2020). Since the N2P2 amplitude is positively correlated with both the physical and subjective intensities of the noxious stimulus (García-Larrea *et al.*, 1997; Iannetti *et al.*, 2005; Kramer *et al.*, 2016), an optimism effect, i.e. a dampening of pain processing, could also be mirrored in a lower amplitude of nociceptive brain potentials.

The SSR reflects changes in the electrical potential of the skin caused by autonomic activation of the sweat glands (Jörg and Boucsein, 1998; Vetrugno *et al.*, 2003). The SSR is a somato-sympathetic reflex consisting of spinal and subcortical components (Vetrugno *et al.*, 2003) and therefore likewise belongs to immediate responses, which can be activated by nociceptive stimuli. While the effect of optimism on the SSR during pain has so far not been examined, optimism has been associated with a weakened reaction of the autonomic nervous system in a prior study measuring cardiac responses as pain-related outcomes (Geers *et al.*, 2008). It can therefore be hypothesized that the SSR is likewise reduced by an optimism induction.

To examine whether there is a selective effect of optimism on the perception of painful, rather than heat stimuli in general, both non-painful and painful heat stimuli are used. We expect that optimism impacts only or at least more strongly on painful stimuli.

Our hypotheses are as follows: first, we hypothesize that induced optimism leads to lower amplitudes of both early CHEPs and the SSR during phasic painful heat stimuli. Second, we hypothesize that induced optimism leads to lower self-report pain ratings of these stimuli.

## Methods

### Participants

A total of 47 healthy, pain-free individuals participated in the current study. Of them, 57% were women (*n* = 27). Age ranged from 18 to 35 years [mean (±s.d.) age: 23.13 (±3.75) years]. The sample size was computed in G*Power based on the literature-based assumption of moderate effect sizes of psychological variables on pain responses (Thorn *et al.*, 2004; George *et al.*, 2007). The following parameters were entered in G*Power: statistical test = repeated measures analysis of variance (ANOVA), within-between factor interaction, alpha level = 0.05, estimated effect size = 0.25, number of groups = 2, number of measurements = 2 and correlation among repeated measures = 0.60. Participants were recruited via advertisements and mailing lists in the local university (Bamberg, Germany). The exclusion criteria were current experience of acute or chronic pain, psychological or physical illnesses, pregnancy or pain-influencing medication. These were assessed via self-report. Participants were asked not to take alcohol or other psychotropic drugs 24 h before the experiment, not to consume cigarettes 1 h before the experiment and to postpone the appointment if the participants developed clinical pain or if any central nervous system-relevant medication had to be taken on that day. All participants provided informed consent and received course credits or monetary compensation. The study protocol was approved by the ethics committee of the University of Bamberg (Bamberg, Germany).

### Procedure

#### *General protocol (* *Figure 1*)

The experiment consisted of one laboratory session. After providing informed consent and demographic information (age, sex; use of contraception and phase of the menstrual cycle for women) and filling out the trait questionnaires (Pain Catastrophizing Scale: ‘PCS’, Life Orientation Test-Revised Version: ‘LOT-R’), the electrodes for the CHEPs and the SSR were attached. Having received instructions, participants practiced giving their ratings on five stimuli with temperatures of either 45°C or 51°C in a randomized order. After these trials, the first block containing 40 stimuli of either 45°C or 51°C (see later for details) was administered. Directly afterwards, the state questionnaires ‘Future Expectancies Scale’ (FEX; Hanssen *et al.*, 2013) and the 'Positive and Negative Affect Schedule’ (PANAS; Watson *et al.*, 1988), which served as a manipulation check to register changes in affect and situational optimism, were filled out for the first time (pre-measurement). Subsequently, the experimental manipulation (‘BPS’ or ‘Typical Day’ (TD) imagery and writing task) was performed. After filling out the ‘FEX’ and ‘PANAS’ for a second time (post-measurement), participants received the second block of painful stimulation consisting again of 40 stimuli. During both blocks of stimulation, CHEPs, SSR and self-report pain ratings were recorded. After roughly 2 h with intermittent breaks, the participants were thanked and debriefed, and the session was concluded.

**Fig. 1.**
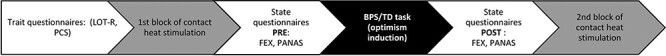
General protocol of the experiment.

#### Experimental manipulations

##### Optimism induction.

Situational optimism was induced with the BPS manipulation, a positive future-thinking technique based on work by King (2001), which has been repeatedly and successfully used before in experimental studies (Peters *et al.*, 2016; Traxler *et al.*, 2019; Basten‐Günther *et al.*, 2021). Participants were instructed to carry out a writing and imagery exercise. In a randomized order, half of the participants were assigned to the BPS condition (*n* = 24), which required them to write about their life in the future where everything had turned out for the best. The other half of the participants were assigned to the control condition (*n* = 22), whose task consisted of writing about a TD. The instructions for BPS and TD were as follows (Sheldon and Lyubomirsky, 2006):

- BPS condition:

Thinking about your best possible self means that you imagine yourself in the future, after everything has gone as well as it possibly could. You have worked hard and succeeded at accomplishing all the goals of your life. Think of this as the realization of your dreams, and that you have reached your full potential.

- TD condition:

Thinking about your typical day means that you take notice of ordinary details of your day that you usually don’t think of. These might include particular classes or meetings you attend to, people you meet, things you do, typical thoughts you have during the day. Think of this as moving through your typical day, hour after hour.

Both manipulations had the same procedural format: participants were requested to think for 1 min about what to write, then to write uninterrupted for 15 min, followed by 5 min of imagining the story they had just been writing. Instructions were given both verbally and in writing. The manipulation check was followed immediately by asking the participants to fill out the FEX and PANAS for a second time to register changes in situational optimism and affect and to answer three questions about the valence, vividness and positivity of their imaginations [‘Quality of Imagery’ (QoI); Peters *et al.*, 2010] to check whether the imagination task was carried out successfully.

##### Pain induction.

Contact heat evoked brain potentials (CHEPs) and skin potentials (SSR) were elicited with a CHEP sensory and pain evaluation stimulator (CHEPS; Medoc, Israel). Participants received 40 phasic heat stimuli per block, which had fixed temperatures of 45°C for 50% of the stimuli and 51°C for the other 50% (a baseline temperature of 35°C, a plateau duration of 10 ms, a rate of rise/fall of 70°C/40°C per second and an interstimulus interval of 15 s). In accordance with prior studies, the 45°C stimuli were expected to be perceived as non-painful but still clearly distinguishable from the baseline temperature and to elicit the same types of evoked potentials (N2 and P2) as painful stimuli, albeit with a smaller amplitude (Granovsky *et al.*, 2008). The 51°C stimuli were expected to be rated as painful (Le Pera *et al.*, 2002; Granovsky *et al.*, 2008; Roberts *et al.*, 2008; Campbell *et al.*, 2010). Participants were instructed that there were going to be both ‘weak’ and ‘strong’ stimuli. They were thus not aware that there were only two different intensities. The sequence of the 45°C and 51°C stimuli was randomized once and then set for all participants. Rules for randomization were that per 10 stimuli there had to be five painful and five non-painful stimuli. Each set of 10 stimuli was randomized separately by drawing the order of 10 pieces of paper, five of which were labelled with ‘painful’ on the inside, five labelled with ‘non-painful’. All stimuli were applied to participants’ volar part of the left forearm (2–4 cm beneath the cubital joint) with a round surface stimulator of a 27 mm diameter. The thermode was held by the experimenter, allowing for flexible placement to prevent receptor fatigue and peripheral habituation. It was moved several millimetres after each stimulus so that the overlapping positions described a full circle across the upper inner part of the forearm during each block of stimulation (Priebe *et al.*, 2016). 2000 ms before each stimulus, a small light located in front of the participant lit up for 500 ms to indicate the imminent stimulus onset.

### Measures

#### Contact heat evoked potentials

Electroencephalography (EEG) recording drew upon the guidelines proposed by Keil *et al.* (2014). Recording was accomplished by a BrainAmp DC amplifier (Brain Products GmbH, Germany) with a sampling rate of 500 Hz and a recording bandwidth from 0.15 to 100 Hz. For electrode placement, a commercial cap with 20 tin electrodes (Electro-Cap International Inc, Eaton, OH, USA) realizing the international 10–20 system (Silverman, 1963) was used. For our study, we recorded from Cz (central, reference), Pz (parietal) and Fz (frontal). Further electrodes were placed on the mastoids (A1, A2) for offline re-referencing the data in order to regain Cz. The ground was placed between Fz and Fpz. In addition, tin electrodes were placed above and below the right eye for a vertical electrooculogram (EOG) and on the outer canthi for a horizontal EOG to correct evoked potentials for eye movements and blinks. To keep the impedances low, the respective skin parts were cleansed with ethanol, dead skin cells were removed and a gel for improving electrical connectivity (Electro-Cap International Inc, USA) was applied. Participants were instructed to move as little as possible and keep a relaxed sitting position. As soon as impedances on the EEG recordings were <5 kΩ for all EEG electrodes and <8 kΩ for all EOG electrodes, and the signal seemed free of disturbances by movements, the stimulation protocol was started.

##### Data parameterization.

To determine N2 and P2 amplitudes as well as N2P2 peak-to-peak amplitudes, EEG data were analysed offline in the BrainVision Analyzer (Brain Products, Germany) following the protocol described by Granovsky *et al.* (2008) and Priebe *et al.* (2016). Data were segmented from 100 ms before to 2000 ms after stimulus onset. The 100 ms before stimulus onset was used for baseline correction. Automatic and manual artefact correction tracked all potentials with amplitudes less than −50 μV and more than +50 μV, with differences of >50 μV between two consecutive sampling points or with differences of >100 μV between the most positive potential and the most negative potential. Segments with artefacts were excluded from all analyses. Only data from Cz were analysed as N2P2 has been shown to be highest at this site (García-Larrea *et al.*, 1997). For each block, averages were calculated for the 20 potentials evoked by the 45°C stimuli as well as for the 20 potentials evoked by the 51°C stimuli. If <70% (i.e. 14 out of 20) of the trials per stimulus category (45°C or 51°C) were free from artefacts, a missing value was entered for this stimulus category. The averaged signals were used to determine two components: based on the study by Priebe *et al.* (2016), N2 was defined as the most negative peak in a time window from 200 to 500 ms; P2 was defined as the most positive peak in a time window from 400 to 650 ms. As latencies of these peaks may slightly vary depending on different factors such as the heating rate of the stimulator or the distance of the site of stimulation from the brain, time windows were chosen a little more liberally than with Granovsky *et al.* (2008). Visual and statistical inspection confirmed that peaks in our data occurred during the chosen time windows (N2: average 353.43 ms; P2: average 523.18 ms) and that latencies were comparable to those found in prior studies. For further analyses, the peak-to-peak N2P2 amplitude, i.e. the absolute difference between the voltage of the N2 and the P2, was calculated. Consequently, four N2-, P2- and N2P2-difference amplitude scores resulted for each subject (45°C pre, 45°C post, 51°C pre and 51°C post). N2 and P2 latencies were not kept for further analyses as stimulus intensity has been shown to be associated with N2 and P2 amplitudes, but not with their latencies for other pain modalities (Iannetti *et al.*, 2005).

#### Sympathetic skin response

SSRs were assessed by use of the SUEmpathy100 (SUESS Medizintechnik, Germany). The skin was prepared as described with the CHEPs. Afterwards, the measurement electrode was fixed on the thenar eminence of the right hand, and the reference electrode was fixed on the proximal third of the right forearm (Jörg and Boucsein, 1998). The ground electrode was fixed on the lateral part of the right elbow. The biosignal was sampled at a rate of 512 Hz.

Following the protocol described in detail by Dittmar *et al.* (2015), SSR data were analysed in order to determine the peak-to-peak amplitude between the first negative peak (N1) and the subsequent positive peak (P1), i.e. the N1P1-difference amplitude (Kucera *et al.*, 2004). To be valid, an N1P1 complex had to show a first deflection from zero before 2.1 s and a latency of P1 <6 s. The amplitudes of all valid stimuli were averaged for each category of stimulus intensity (45°C pre, 45°C post, 51°C pre and 51°C post). Thus, four N1P1-difference amplitude values resulted for each subject. In case of <70% (i.e. 14 out of 20) of valid trials in a category, a missing value was entered for this category.

#### Self-report pain ratings

Participants were instructed to rate the perceived painfulness of each stimulus by answering two questions after each stimulus: (I) was this stimulus painful? (II) How painful was this sensation? For the first question, a dichotomous answer (yes or no) had to be given. For the second question, participants had to choose the respective category from an 11-point numeric rating scale (NRS) ranging from 0 (‘not painful at all’) to 10 (‘most intense pain imaginable’). Answers were given orally, recorded and transcribed after the experiment. Participants were asked to give their rating without request after they had sensed the stimulus but were reminded by the experimenter if necessary.

#### Questionnaires

##### Trait questionnaires

###### Life Orientation Test-Revised

The validated German version (Krohne *et al.*, 1996) of the LOT-R (Scheier *et al.*, 1994) was used to assess the level of dispositional optimism. The LOT-R has 10 items that are rated on a 5-point Likert scale, ranging from 0 (‘strongly disagree’) to 4 (‘strongly agree’). There are three positively phrased items, three negatively phrased items and four filler items. A total score was calculated after reversing the negatively phrased items.

###### Pain Catastrophizing Scale.

A German translation (Meyer *et al.*, 2008) of the PCS (Sullivan *et al.*, 1995) was used to assess habitual catastrophic thinking related to pain. The PCS has been widely used in research on pain catastrophizing (Wheeler *et al.*, 2019). Participants are instructed to reflect on thoughts or feelings during past painful experiences. The scale comprises 13 items that are rated on a 5-point scale, with the end-points ‘not at all’ and ‘all the time’.

##### State questionnaires

###### Future Expectancies Scale.

The FEX (Hanssen *et al.*, 2013) was administered to assess state optimism. A German translation of the questionnaire was used, which has been translated in a standard ‘forward-backward’ procedure and used in prior studies by the authors (Peters *et al.*, 2016; Basten‐Günther *et al.*, 2021). The FEX consists of 10 statements describing a positive future event and 10 statements describing a negative future event. Participants rate the likelihood that they will experience each specific event on a 7-point Likert scale, ranging from 1 (‘not at all likely to occur’) to 7 (‘extremely likely to occur’). The FEX has previously been demonstrated to be responsive to the ‘BPS’ optimism manipulation (Hanssen *et al.*, 2013; Boselie *et al.*, 2014; Basten‐Günther *et al.*, 2021). The subscores FEX positive and FEX negative were used for further analyses.

###### Positive and Negative Affect Schedule.

Mood was assessed with the PANAS (Watson *et al.*, 1988). The PANAS consists of 10 items measuring positive affect and 10 items measuring negative affect. Participants indicate the degree to which a certain feeling is present at that moment on a 5-point Likert scale ranging from 1 (‘not at all’) to 5 (‘extremely’). The subscores PANAS positive affect (PANAS_PA) and PANAS negative affect (PANAS_NA) were used for further analyses. For the PANAS, a validated German version was available (Glaesmer *et al.*, 2008), which has repeatedly been used before (Peters *et al.*, 2016; Basten‐Günther *et al.*, 2021).

###### Quality of Imagery:

Two 10 cm visual analogue scales (VASs) were used to rule out qualitative differences in participants’ imagery between the BPS group and the TD group. Participants were asked ‘How well could you imagine yourself in the situation you described in your writing?’ (not at all to extremely well) and ‘How vivid were the pictures you imagined?’ (not vivid at all to very vivid). A third 10 cm VAS was administered to determine whether imagery in the BPS group was more positive than in the TD group: ‘How negative or positive were your imaginations?’ (very negative to very positive). These questions have been used before with the BPS paradigm (Hanssen *et al.*, 2013; Basten‐Günther *et al.*, 2021).

### Statistical analyses

#### Manipulation check

To control whether the optimism induction was successful (manipulation check), four separate 2 × 2 repeated measures ANOVAs were computed. The PANAS and FEX subscales served as dependent variables, while the experimental condition was entered as the fixed factor. These ANOVAs were meant to analyse differences between the pre- and post-measurement, i.e. to detect changes in state affect and optimism induced by the experimental manipulation.

Independent samples *t*-tests were used to investigate group differences in each of the three scales of the ‘QoI Questionnaire’.

To check the efficacy of the pain manipulation, chi-squared tests were applied to compare the frequency by which the 45°C stimuli and the 51°C stimuli were rated as painful, respectively. Besides, the main effect of stimulus intensity was tested in the analysis of covariance, which will be described in the following section.

#### Effect of optimism induction

CHEP data (N2P2-difference amplitudes and N2 and P2 amplitudes) and SSR data (N1P1-difference amplitudes), as well as the self-report pain ratings, were subjected to separate 2 × 2 × 2 repeated measures ANOVAs with the between-factor experimental condition (BPS *vs* TD) and the within-factors time of measurement (pre *vs* post) and stimulus intensity (45°C *vs* 51°C). According to our hypotheses, we expected a time × condition × intensity interaction effect showing a significant influence of the optimism induction on the outcome variables during truly painful stimuli (51°C).

All analyses were conducted with SPSS 24, and the alpha level was 0.05 (α) throughout.

## Results

### Descriptive statistics

Data of one participant who aborted the experiment during the TD imagery and writing task due to acute headaches were excluded. Thus, data of 46 participants (BPS: *n* = 24; TD: *n* = 22) were included in the analyses. Means and standard deviations for demographic variables, dispositional optimism (‘LOT-R’) and dispositional pain catastrophizing (‘PCS’) are shown in Table 1.

**Table 1.** Demographics and trait measures of optimism and pain catastrophizing—BPS, treatment group; TD, control group

	TD			BPS		
	*n = *22			*n = *24		
Sex: female	12 (54.5%)			14 (58.3%)		
	Mean (s.d.)	Min.	Max.	Mean (s.d.)	Min.	Max.
Age (years)	23.36 (3.36)	18	31	23.00 (4.18)	18	35
LOT-R	17.86 (2.75)	13	23	17.88 (3.60)	8	23
PCS	18.45 (6.03)	2	29	18.92 (6.14)	10	30

In the TD group, 54.5% (*n* = 12 out of 22) of participants were female, and in the BPS group, 58.3% (*n* = 14 out of 24) were female.

### Manipulation check: optimism induction

To control whether the optimism induction was successful, state measures of optimism and affect were analysed.

#### Future Expectancies Scale

**Fig. 2.**
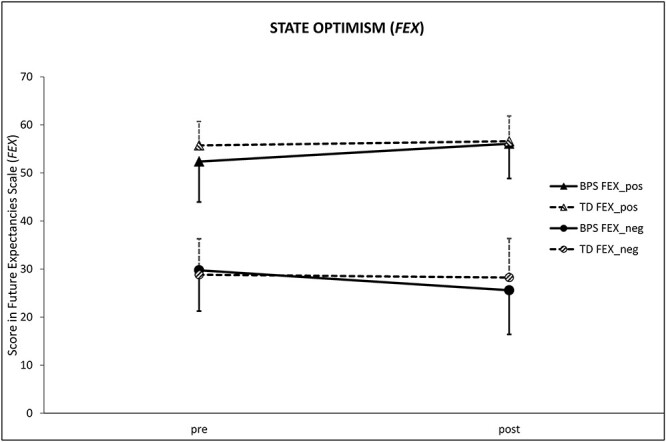
Mean of state optimism (FEX_pos and FEX_neg) at baseline (pre) and after the experimental manipulation (post). A significant time × group interaction effect was found for both FEX subscales. Error bars = +1s.d. (TD)/−1s.d. (BPS). BPS, optimism group; TD, control group.

The significant time** × **group interaction effect, which we expected in the 2 × 2 repeated measures ANOVAs, was found for the FEX subscale negative future expectancies [*F*(1, 44) = 9.42, *P *= 0.004, η*p*^2^ = 0.18], but not for the subscale positive future expectancies [*F*(1, 44) = 7.15, *P *= 0.01, η*p*^2^ = 0.14]. As shown in Figure 2, negative future expectancies (FEX_neg) remained constant in the TD group from the first assessment to the second assessment [*t*(21) = 0.62, *P* = 0.54], whereas the BPS group scored significantly lower after than before the optimism induction [*t*(23) = 4.77, *P* < 0.001]. In the positive expectations subscale, the BPS group significantly increased from the pre- to the post-measurement [*t*(23) = 4.11, *P* < 0.001], while the TD group’s level remained constant [*t*(21) = 0.62, *P* = 0.54].

Given these significant decreases in negative future expectancies after the BPS task, we can assume that the optimism manipulation was successful. This is in accordance with prior studies using the same paradigm (Peters *et al.*, 2010, 2016; Hanssen *et al.*, 2013; Basten‐Günther *et al.*, 2021).

#### Positive and Negative Affect Schedule

**Fig. 3.**
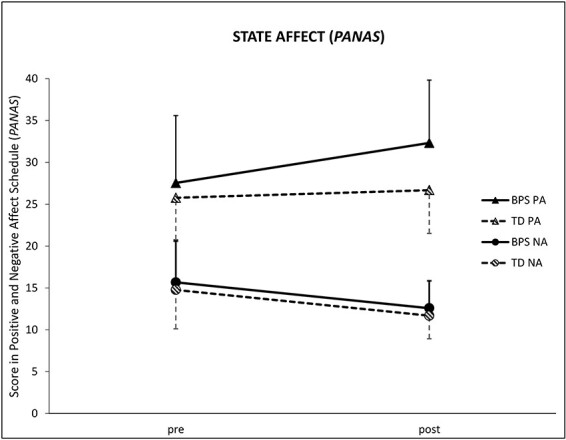
Mean of PANAS state positive/negative affect (PA and NA) at baseline (pre) and after the experimental manipulation (post). Error bars = +1s.d. (BPS)/−1s.d. (TD). BPS, optimism group; TD, control group.

State positive affect measured by the PANAS was influenced by the optimism induction as there was a significant time** × **group interaction effect for the subscale positive affectivity [*F*(1, 44) = 4.13, *P *= 0.048, η*p*^2^ = 0.09]. As indicated by the *post hoc t*-tests, the groups did not differ in the pre-assessment [(*t*(44) = 0.092, *P* = 0.36], while in the post-assessment, the BPS group scored significantly higher than the TD group [*t*(44) = 2.57, *P* = 0.01]. The BPS group’s score significantly increased from the pre- to the post-measurement [*t*(23) = 4.34, *P* <0.001], while the TD group’s score remained constant [*t*(21) = 0.06, *P* = 0.57]. For the subscale PANAS negative affectivity, no time** × **group interaction effect was found [*F*(1, 44) = 0.00, *P *= 0.995, η*p*^2^ = 0.00] (Figure 3).

#### Quality of Imagery

The BPS and TD groups did not significantly differ in the VAS about the success [*t*(44) = 1.39, *P* = 0.17] and the vividness [*t*(44) = 0.64, *P* =0.59] of their imaginations, which were assessed after the imagery/writing task. On the third question about the positivity/negativity of the imaginations, the BPS group scored significantly higher than the TD group [*t*(44) = 3.15, *P < *0.01]. The observed differences between the groups in terms of state optimism therefore seem to be selectively due to a more positive content in the writing and imagery on part of the BPS group. This is in line with the aims of the BPS paradigm and further corroborates the assumption that our optimism manipulation was successful.

### Effect of optimism induction: CHEPs (Figure 4)

CHEP data of 39 participants (BPS: *n* = 21, TD: *n* = 18) were valid (i.e. comprising <30% of trials per category with artefacts, as described in the Methods section) and could thus be included in the analyses. EEG plots are shown in Figure 5.

**Fig. 4.**
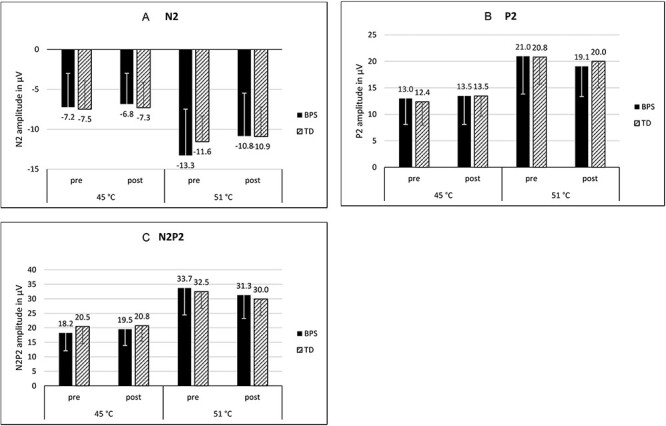
Mean amplitude of the CHEPs depending on stimulus intensity, experimental condition and time of measurement. (A) N2 component, (B) P2 component and (C) N2P2 complex. Error bars = +1s.d. (N2)/−1s.d. (P2, N2P2). BPS, optimism group; TD, control group.

**Fig. 5.**
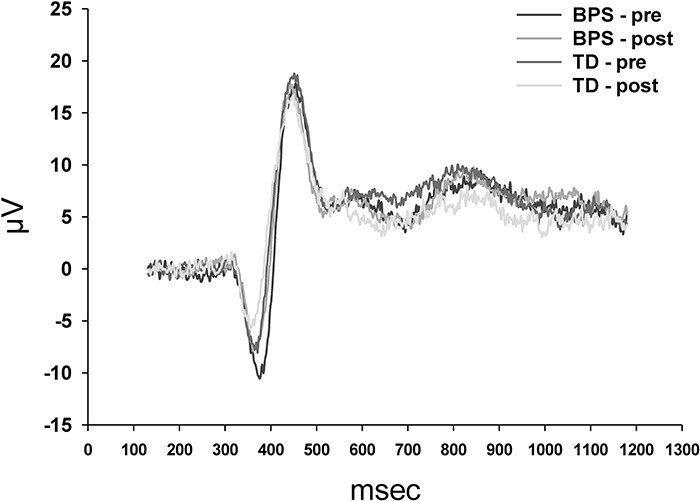
CHEPs pre and post the induction of optimism (BPS) and in a control condition (TD) evoked by noxious heat (51°C/45°C). As can been seen, there were no differences between pre and post and between conditions (BPS/TD). The number of valid subjects was 39.

#### N2

Analyses of the amplitude of the N2 did not reveal a significant time** × **condition** × **intensity interaction effect [*F*(1, 37) = 0.69, *P* = 0.41, ηp^2^ = 0.02], implying that there was no selective effect of the optimism induction on the N2 during painful stimuli. Validating our stimulus intensity manipulation, the N2 was significantly more pronounced in the 51°C stimuli than in the 45°C stimuli [main effect of temperature: *F*(1, 37) = 95.52, *P* ≤ 0.001, η*p*^2^ = 0.72].

#### P2

In the amplitude of the P2, there was no significant time** × **condition** × **intensity interaction [*F*(1, 37) = 0.55, *P* = 0.46, η*p*^2^ = 0.02], meaning that there was no selective effect of the optimism induction on the P2 during painful stimuli. As with the N2, there was a significant main effect of temperature [*F*(1, 37) = 124.75, *P* ≤ 0.001, η*p*^2^ = 0.77].

#### N2P2

In the N2P2 complex, no significant time** × **condition** × **intensity interaction effect was found [*F*(1, 37) = 0.002, *P* =0.96, η*p*^2^ ≤ 0.001]. There was thus no selective effect of the optimism induction on the N2P2 during painful stimuli. The N2P2 was significantly larger in the 51°C stimuli than in the 45°C stimuli [main effect of temperature: *F*(1, 37) = 157.24, *P* ≤ 0.001, η*p*^2^ = 0.81].

### Effect of optimism induction: SSR (Figure 6)

**Fig. 6.**
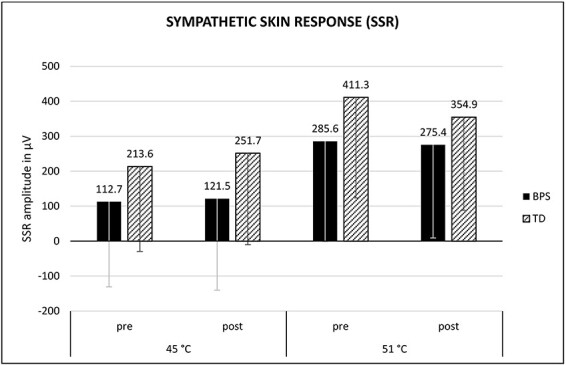
Mean amplitude of the N1P1 complex (SSR) depending on stimulus intensity, experimental condition and time of measurement. Error bars = −1s.d. BPS, optimism group; TD, control group.

For the SSR, data of 42 participants (BPS: *n* = 22, TD: *n* = 20) were valid (i.e. comprising <30% of trials per category with artefacts, as described in the Methods section) and could thus be included in the analyses. No significant time × condition × intensity interaction effect was found [*F*(40, 1) = 2.61, *P *= 0.11, η*p*^2^ = 0.06] (Figure 6). This means that the optimism induction did not selectively alter the SSR during painful stimuli. The SSR amplitude was significantly bigger in the 51°C stimuli than in the 45°C stimuli [main effect of temperature: *F*(40, 1) = 59.49, *P *≤ 0.001, η*p*^2^ = 0.60]. Although the SSR was slightly bigger in the control group compared to the optimism group, this difference was non-significant [main effect of condition: *F*(40, 1) = 2.238, *P *= 0.14, η*p*^2^ = 0.05].

### Effect of optimism induction: self-report pain ratings (Figure 7)

Dichotomous pain ratings (BPS: *n* = 24, TD: *n* = 22) significantly differed depending on stimulus intensity (*χ*^2^(1) = 8.00, *P *= 0.005). The 45°C stimuli were judged as non-painful with an average relative frequency of 0.42, compared to only 0.02 with the 51°C stimuli. The 51°C stimuli were thus very consistently perceived as painful.

**Fig. 7.**
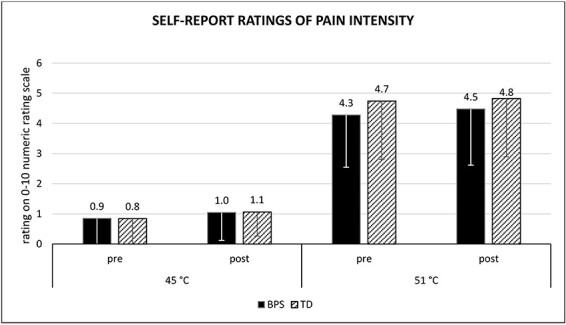
Mean ratings of pain intensity depending on stimulus intensity, experimental condition and time of measurement. Error bars = −1s.d. BPS, optimism group; TD, control group.

As concerns self-report pain ratings on the NRS (BPS: *n* = 24, TD: *n* = 22), there was no significant time × condition × intensity interaction effect [*F*(1, 44) = 0.45, *P *= 0.43, η*p*^2^ = 0.02], meaning that the optimism induction apparently did not influence how intense the painful stimuli were experienced (Figure 7). The 51°C stimuli were perceived as significantly more painful than the 45°C stimuli [main effect of temperature: *F*(1, 44) = 289.00, *P* ≤.001, η*p*^2^ = 0.87]. Ratings of the painful stimuli correlated weakly to strongly (*r* between 0.1 and 0.4) with the N2, P2 and N2P2 components at the corresponding times of measurement (pre and post).

## Discussion

In the present study, the effect of experimentally induced optimism on the earliest components of the response to contact heat stimulation was examined. While there is evidence that the manipulations of pain and optimism were successfully applied, our hypotheses were not confirmed. Consistently, all measured pain outcome variables (N2, P2 and N2P2 amplitudes of CHEPs; N1P1 amplitude of the SSR; self-report pain intensity ratings) were not responsive to the optimism induction.

### Optimism effect on early components of the pain response

To begin with, none of the early pain outcome variables measured in this study was influenced by the optimism induction. As elaborated in the introduction, these early components mainly reflect the processing of the sensory dimension of pain, i.e. of pain intensity. Therefore, our results indicate that induced optimism might not act on the immediate experience of pain intensity. It is of note that evoked potentials and the SSR are well-established psychophysiological pain measures that provide a high degree of objectivity as they are much less prone to individual cognitive biases than, for example, self-report ratings (Huang *et al.*, 2004; Granovsky *et al.*, 2008). Therefore, the fact that no optimism effect was found in these measures—while, in contrast, a clear reaction to the manipulation of stimulus intensity could be seen—underlines and in some way objectifies and validates findings on ‘subjective’ pain measures like self-report ratings, which were likewise unaffected by the optimism manipulation.

It is likely that optimism impacts differentially on different stages in pain processing: According to the four-stage model of pain processing described in the introduction (Price, 1988; Wade *et al.*, 1992), the first, i.e. earliest, stage in pain processing comprises the sensory-discriminative dimension which includes spatial, temporal and intensive features of the sensation. In the second stage, immediate unpleasantness (Wade *et al.*, 1996) is added. It has been shown that the first stage is not at all and the second stage only modestly influenced by individual beliefs, attitudes and reflections on pain. These exert a much bigger influence on the third stage (suffering) and the fourth stage (pain behaviour) (Harkins *et al.*, 1989; Wade *et al.*, 1992). More recent research confirms that early pain components as the P2 and N2 reflect the encoding of sensory aspects of pain (García-Larrea *et al.*, 1997; Iannetti *et al.*, 2005) in a ‘first-order’ nociceptive network (García-Larrea and Peyron, 2013), while appraisal processes have not yet started at that point of time (Wager *et al.*, 2006). Therefore, the effect of expectations and the assignment of meaning are only or at least more strongly reflected with later components of nociceptive processing (Zaslansky *et al.*, 1996; Wager *et al.*, 2004, 2006). Our finding that consistently—across all measured parameters—reactions at early stages of pain processing occurred unchanged despite the successful optimism induction is in line with this model. It provides more evidence that pain processing at early stages is not influenced by elaborate cognitive processes and characteristics as, in our case, optimistic thinking. Since the immediate responsivity of the nociceptive system was not influenced by optimism in our study, it could be worth re-examining whether significant effects of optimism on pain found in other studies reflect later stages of pain processing. This will be discussed in the following.

#### Comparison with studies demonstrating positive effects of optimism on pain

To explain the discrepancies between our findings and those of prior studies suggesting positive effects of optimism on pain ratings and autonomic pain responses, it could be helpful to examine the time frames of stimulation and response measurement.

##### Autonomic pain responses.

While no prior evidence on cortical evoked potentials has to our knowledge been available, there is a study on sympathetic nervous system activity that shows a dampening influence of dispositional optimism on cardiac responses during a 2 min cold pressor task (Geers *et al.*, 2008). The fact that this study used the average heart rate and blood pressure during a tonic pain stimulation further corroborates our assumption that optimism only acts on later stages of pain processing which would be tapped by a measure sampling data over 2 min, while in our experiment, a potential optimism influence had not yet unfolded and was therefore not reflected in the SSR as an early pain component. As vegetative pain components are seen as part of or at least closely linked to affective pain responses (Dubé *et al.*, 2009; Boettger *et al.*, 2010), an influence of optimism on later autonomic activity via appraisal and coping processes, which have an impact on pain-related affect (Park and Sonty, 2010; Gruszczyńska and Knoll, 2015), would be plausible and should be examined in further studies.

##### Self-report pain ratings.

For self-report ratings, a similar interpretation might apply: as with the SSR, no optimism effect on ratings of pain intensity was found, which is opposite to one study showing a significant influence of the BPS exercise on self-report ratings (Hanssen *et al.*, 2013). Given the successful optimism manipulation, one could therefore assume that, as argued earlier, the verbal reaction to the short, 10 ms pain pulses that we applied—although retrospectively assessed several seconds after the CHEPs—still reflects early stages of pain processing (stage 1 or 2 according to Price, 1988) when cognitive influences related to optimism are still not likely to have an effect. The fact that Hanssen *et al.* (2013) found a pain-dampening effect of optimism during a longer-lasting, tonic pain stimulation, namely a 2 min cold pressor task, and that of the three ratings given at 20, 40 and 60 s, the highest difference between the BPS group and the TD group was measured in the last rating at 60 s of immersion, supports this idea of late effects.

Since several other studies did not find an effect of the BPS task on self-report pain ratings either (Boselie *et al.*, 2014; Traxler *et al.*, 2019; Basten‐Günther *et al.*, 2021), it cannot be ruled out that the effect of this optimism induction on experimental pain either does not exist or is rather weak, which would render significant results unlikely. More research is needed to clarify these speculations.

### Limitations

The present study is to our knowledge the first to examine the effect of an experimental optimism induction on the earliest stages of pain processing by measuring pain-related CHEPs and the SSR. Due to the stimulation procedure, the experimenter was standing next to the participants. For this reaon, it has to be taken into account that the optimism–pain relationship might have been modified by social processes. Although the stimulation arrangement provides high ecological validity by resembling clinical examinations or treatment procedures and although a suchlike influence might be rather weak because participants were asked to focus their attention on the signal light in front of them, it seems nevertheless advisable to test for systematic social influences in future studies. Despite the random allocation of participants to the experimental conditions and although a randomization check was performed for some variables such as, for example, age, sex or the trait questionnaire scores, we cannot rule out that groups may still have differed in other, unknown variables which might have distorted the results. Since our study included only young, healthy participants, the results should be further validated in samples with older adults—in whom there are indications of stronger effects of optimism on pain (Basten-Guenther *et al.*, 2019)—and in clinical populations. As indicated by the manipulation check, our use of the BPS seems to have been effective. Indeed, in a meta-analysis of different optimism-fostering interventions by Malouff and Schutte (2017), the BPS technique has been shown to provoke the strongest increases in participants’ optimism. Nevertheless, it could be interesting to also study other optimism manipulations such as, for example, cognitive-behavioural or mindfulness-based exercises, and to apply interventions repeatedly over a longer period of time.

### Conclusions and outlook

In the present study, the examined early components of the response to painful heat stimuli were consistently not influenced by an optimism induction. Our findings imply that early stages in pain processing, elaborating mainly on the sensory-discriminative dimension and the immediate pain affect (Price, 1988; Wade *et al.*, 1996), are likely not influenced by optimism. Future research could therefore include measures tapping both early and later stages of pain processing, comparing the influence of optimism on the different stages to look for further validation of our results and hypotheses. The present findings could be enlarged by studying other pain outcome variables such as different autonomic responses to pain, by applying different stimulus lengths and pain modalities and by transferring the paradigm to clinical populations.

## Data Availability

The data underlying this article will be shared on reasonable request to the corresponding author.
